# Chronic obstructive pulmonary disease patients' quality of life and its related factors: A cross-sectional study of the Jordanian population

**DOI:** 10.12688/f1000research.121783.1

**Published:** 2022-05-26

**Authors:** Enas A Assaf, Angham Badarneh, Ahmad Saifan, Nabeel Al-Yateem

**Affiliations:** 1 Faculty of Nursing, ​​Applied Science Private University, Amman, 11931, Jordan; 2 Department Of Nursing, - Prince Hamza Hospital, Amman, 11224, Jordan; 3 College of Health Sciences, Department of Nursing, University fo Sharjah, Sharjah, 27272, United Arab Emirates

**Keywords:** Chronic Obstructive Pulmonary Disease, COPD, Quality of Life, QoL, Jordan, Quantitative study, Survey design, Cross-sectional study.

## Abstract

**Background: **Chronic Obstructive Pulmonary Disease (COPD) is the third leading cause of death globally, mostly in low- and middle-income countries. It is estimated that 6.5% of Jordanians under 50 and 37.5% of those over 70 years of age are affected. The country's air pollution levels surpass recommended levels, increasing the disease incidence and burden on individuals and the health system. COPD is a long-term, severe, and exhausting condition. In Jordan, patients are highly dependent and frequent users of the healthcare services; therefore, their Quality of Life (QoL) is highly influenced by the health care they receive. The QoL of COPD patients must be studied to devise interventions that can help patients cope with this disease and for healthcare systems to improve their service.

**Method:** A cross-sectional correlational study of 200 COPD patients. The Arabic WHO Quality of Life Questionnaire Short Form was used to collect data.

**Results:** The mean COPD patient QoL score was 10.66 (SD=1.58), showing poor QoL perception. The physical domain had the lowest perceived QoL (10.232, SD=1.912), while the environmental domain had the highest (10.948, SD=1.636). Unmarried, non-smokers, and employed had better QoL (M=11.04, M=10.92, M=12.04). Age categories 50-61 exhibited greater mean QoL than age category 61 or higher (M=11.44, M=10.84, M=10.08). Private health services are characterized by short waiting times, availability of different diagnostic and treatment services, and skilled staff was related to better QoL.

**Conclusions:** QoL for COPD patients seems to be an area requiring urgent attention from Health service providers and planners. Patients should be adequately supported and cared for to have a good QoL. In Jordan, COPD patients' QoL is highly influenced by lack of physical activity, emotional distress, and anxiety. Therefore, better health care services is needed to address all these areas adequately.

## Introduction

Chronic Obstructive Pulmonary Disease (COPD) is prevalent worldwide. The World Health Organization has reported the disease as the third leading cause of death globally, causing 3.23 million deaths in 2019, with Over 80% occurring in low- and middle-income countries (LMIC). ^1^ In Jordan, COPD has a prevalence rate of 6.5% in patients under 50 years of age and 37.5% in patients aged ≥70 years, which is much higher than the reported international prevalence rate, especially for the latter age group. ^2^

COPD causes persistent and progressive respiratory symptoms, including difficulty in breathing, cough, and thick viscous mucus secreted within the respiratory passages. It also exacerbates during physical exercise and exertion. ^3^^–^^6^ It is usually caused by prolonged exposure to dangerous chemicals and particles and by individual variables such as early experiences that affect lung development and heredity. Tobacco smoke exposure, indoor air pollution, occupational dust, gases, and chemicals all contribute significantly to the chance of developing COPD.

Early diagnosis and treatment, especially assistance for smoking cessation, are necessary to slow the progression of symptoms and minimize flare-ups. ^7^ As COPD advances, patients increasingly struggle to do routine everyday activities, frequently due to dyspnea. In addition, since the COVID-19 pandemic, there may be a significant financial burden because of the reduced workplace and home productivity and medical care costs. During flare-ups, patients with COPD may notice an increase in their symptoms which may require further treatment at home or admission to the hospital for emergency care, as severe flare-ups can be fatal. ^7^

There is an association between COPD and many other diseases, such as cardiovascular, lung cancer, osteoporosis, skeletal muscle, cachexia, gastrointestinal, metabolic, other respiratory illnesses, and mental health issues such as anxiety and depression. ^8^ The correlation between COPD symptom load and anxiety and depression is important, as the combination of these disorders can worsen the disease course, duration, and outcome. ^9^

Studies have shown that COPD negatively correlates with the Quality of Life (QoL), ^10^^–^^17^ and this correlation worsens as the severity increases. Kushwaha *et al.* (2020) reported “impaired” life processes and health-related quality of life among patients with COPD. ^18^^,^^19^

In Jordan and in the Middle East region, there is a lack of national or regional studies about the extent of the COPD disease in the country and the service offered for these patients. Most studies and evidence come from developed countries. Also, available epidemiological data significantly underestimate the entire frequency of COPD because the disease is typically not identified until it is clinically evident and moderately progressed. ^20^^–^^22^ Furthermore, the causing and the exacerbating factors for COPD are also common in Jordan; for example, environmental toxins, pollutants, smoking habits, and occupational chemicals exposures that are trigger for respiratory illnesses have been reported to be much higher than the accepted international standards. ^23^^–^^25^

A recent study in Jordan ^26^ investigated uncertainty, anxiety, and the Health-Related Quality of life (HRQoL) among COPD patients and found higher levels of these variables among their study participants. Although important, the finding lacked details about these issues since the HRQoL was one variable measured in the study, among others.

COPD is a long-term, severe, and exhausting condition. Patients are highly dependent and frequent users of the healthcare services; therefore, their Quality of Life (QoL) is an important consideration to measure and improve. In addition, their QoL is expected to be highly influenced by the health care services they receive. In Jordan, the QoL of patients with COPD must be studied to devise interventions that can help patients cope with this disease and for healthcare systems to improve their services.

Therefore, this study will focus on QoL and assess its different domains such as physical, psychological, social interaction, and environmental. Additionally, this study will help identify the concerns of COPD patients about their QoL; and the main determinant factors affecting it.

## Study objective

The objective of this study is to assess chronic obstructive pulmonary disease patients’ quality of life and its related factors within the Jordanian population.

## Methods

### Study design and setting

The study design was cross-sectional correlational. The study was conducted in the outpatient clinics in four hospitals in Jordan, from different sectors (public, private and educational) and in different cities (i.e., Amman, Irbid, Zarqa). The study was conducted between April 2021 and May 2021.

This study included 200 participants. All COPD patients aged 18 and over who attended the thoracic clinic were invited to the study. The exclusion criteria for participation were patients with other comorbidities, and individuals with mental health problems that prevent them from consenting to participitation in the study.

### Study sample and sample size

The sample size was calculated using the G*power software version 3.1 (RRID:SCR_013726) based on the following parameters: ANOVA test, alpha 0.05, the medium effect size of 0.25, power of 0.8, number of groups 4. The included sample size (n=200) was enough to achieve these parameters.

### Variables and measurement

A self-administered questionnaire was used in this study; the questionnaire consisted of two parts: social-demographic questions (10 questions) and the Arabic World Health Organization QoL Instrument (Arabic -WHOQoL-BREF ^27^) (See Underlying data). ^28^ The tool included 26 Likert-type questions with answers ranging from 1 (disagree/not at all) to 5 (completely disagree/extremely). The questions assessed an individual’s perceptions of his/her well-being and health over the past two weeks. The questionnaire contained four domains which were: physical health (7 items), psychological health (6 items), social relationships (3 items), and environmental health (8 items).

The questionnaire is well-known and widely used and translated into different languages. Moreover, it has been validated with different populations and different illnesses; hence it is useful for cross-cultural and cross-disease comparisons. ^27^^,^^29^^,^^30^

### Data collection procedure

In the outpatient pulmonary clinic, the researcher obtained the list of patients attending the clinic, and patients with COPD were identified by the doctor and were asked to participate in the study. A suitable place was chosen to collect the data in coordination with the head nurse. The participants were given the questionnaires and the needed instructions, and the researcher remained there to answer any questions and collect the filled questionnaires.

### Statistical methods

Descriptive statistics were used to describe sample characteristics, individual items, and the mean scores for the subdomains and the whole scale. Tests of associations, differences, and correlations were also used to assess the relationship or associations between the study variables and compare the sample subgroups’ scores. These tests were selected based on the type of variables and the normality assessment of the continuous variables. The used tests included Chi-Squared tests, t-test, ANOVA tests, Man Whitney, Kruskal Wallace, and Pearson or Spearman Correlation tests. The p-value was set at 0.05.

### Ethical considerations

Approval was obtained from the Institutional Review Board (IRB) of Applied Science Private University (IRB # 2020-2021-2-1) prior to data collection. Patients that agreed to participate in the study gave written informed consent after receiving an explanation of the study’s purposes, duration, risks, and benefits and their role in the study. They were informed that they could withdraw from the study anytime they wanted.

## Results

### The socio-demographic characteristics of the participants

As demonstrated in Table 1, majority of participants were male (n =149, 74.5%), married (n=102, 51.0%), retired (n=147, 73.5%), smoker (n=105, 52.5%), and have university degree (n=176, 88.0%) (See Underlying data). ^28^ The participants in the age group 51-60 years were greater (n=86, 43%) than the age group of ≤50 years (n= 34, 17%), and those ≥61years old (n=80, 40%). Patients with low monthly income were higher (n=113, 56.5%) than those with moderate and high income (n=87,43.5%). Regarding respiratory symptoms, 94% (n=188) had a persistent cough, 2% (n=4) had sputum, 68% (n=136) had wheezing, and 90% (n=180) had shortness of breath. In addition, the duration of COPD ranged from one month to 23 years, with a median of eight years. In this study, 34% of participants (n= 68) were recruited from educational hospitals, 33% (n=66) were recruited from private hospitals and finally, 33% (n=66) were recruited from governmental hospitals.

**Table 1.** Socio-demographic characteristics of the sample (n=200).

	Variable	N (%)
Gender	Female	51 (25.5%)
	Male	149 (74.5%)
Age	≤50	34 (17.0%)
	51-60	86 (43%)
	≥61	80 (40%)
Marital status	Married	102 (51.0%)
	Unmarried	98 (49.0%)
Monthly income	≤499	113 (56.5%)
	≥500	87 (43.5%)
Employment status	Employed	25 (12.5%)
	Not employed	28 (14.0%)
	Retired	147 (73.5%)
Disease symptoms	Cough	188 (94.0%)
	Sputum	4 (2.0%)
	Wheezing	136 (68.0%)
	SOB	180 (90.0%)
Smoking status	Smoker	105 (52.5%)
	Non-smoker	95 (47.5%)
COPDduration	Mean	8.1
	Median	8.0
	Minimum	0.10
	Minimum	23.0
	SD	4.8
Educational level	University degree	176 (88.0%)
	School education	24 (12.0%)
Health sectors	Private	66 (33%)
	Governmental	66 (33%)
	Educational	68 (34%)

SOB: Shortness of Breath; COPD: Chronic Obstructive Pulmonary Disease; SD: Standard Deviation.

### QoL among COPD patients

The results in Table 2 showed that the Mean for the total score of QoL was 10.68 out of a maximum possible score of 20 (SD=1.6). Comparing the four domains of QOL, the environmental domain was the highest with a mean score of 10.96 (SD=1.64), while the physical domain was the lowest with a mean score of 10.24 (SD=1.92) (See Underlying data). ^28^

**Table 2.** Analysis of the WHOQoL-BREF items and dimensions.

Dimensions and questions for each dimension	Mean	SD
**Physical dimension**	**10.232**	**1.912**
To what extent do you feel that physical pain prevents you from doing what you need to do?	10.12	2.804
How much do you need any medical treatment to function in your daily life?	1.7	2.672
Do you have enough energy for everyday life?	10.06	2.404
How well are you able to get around?	10.26	2.932
How satisfied are you with your sleep?	10.1	2.944
How satisfied are you with your ability to perform your daily living activities	10.7	2.232
How satisfied are you with your capacity for work?	10.68	2.408
**Psychological dimension**	**10.904**	**1.896**
How much do you enjoy life?	10.66	2.38
To what extent do you feel your life to be meaningful?	10.2	2.592
How well are you able to concentrate?	9.26	3.368
Are you able to accept your bodily appearance?	10.8	2.504
How satisfied are you with yourself?	11.18	2.7
How often do you have negative feelings such as blue mood, despair, anxiety, depression?	13.32	3.676
**Social dimension**	**10.652**	**1.908**
How satisfied are you with your personal relationships?	11.34	2.684
How satisfied are you with your sex life?	9.72	2.876
How satisfied are you with the support you get from your friends?	10.9	2.888
**Environmental dimension**	**10.948**	**1.636**
How safe do you feel in your daily life?	10.98	2.628
How healthy is your physical environment?	10.98	2.628
Have you enough money to meet your needs?	11.28	2.036
How available to you is the information that you need in your day-to-day life?	10.02	3.00
To what extent do you have the opportunity for leisure activities?	8.66	2.94
How satisfied are you with the conditions of your living place?	11.82	2.512
How satisfied are you with your access to health services?	11.94	2.612
How satisfied are you with your transport?	11.9	2.392

### Socio-demographic correlations with QoL

As shown in Table 3, the t-test has shown a statistically significant mean difference in QoL between smokers (M=10.28, SD=1.44) and non-smokers (M=11.072, SD=1.63) in favor of the non-smokers who had a higher mean (p≤0.001). Similarly, a statistically significant difference was found between unmarried (M=10.92, SD=1.36) and married (M=10.4; SD=1.52) in favor of unmarried participants who had a higher score mean (p=0.024), while the QoL was not statistically significant among the other independent variables (gender, income, educational level).

**Table 3.** Independent t-test result for QoL mean differences based on demographic variables.

	Independent variable	N (%)	Mean (SD)	*P* value
Smoking status	Smoker	105 (52.5%)	10.288 (1.44)	0.000*
	Non-smoker	95 (47.5%)	11.072 (1.628)	
Marital status	Married	102 (51%)	10.412 (1.548)	0.024*
	Unmarried	98 (49%)	10.916 (1.576)	
Gender	Female	51 (25.5%)	10.604(1.424)	0.782
	Male	149 (74.5%)	10.676(1.628)	
Monthly income	≤499	113 (56.5%)	10.392 (1.312)	0.06
	≥500	87 (43.5%)	11.008 (1.816)	
Educational level	University degree	176 (88%)	10.668 (1.52)	0.802
	School education	24 (12%)	10.584 (1.968)	

ANOVA and post hoc test ( *Scheffe*) were conducted to assess the effect of the employment status (employed, not employed, retired) and age (≤50, 51-60, ≥61) on QoL perceptions. Regarding the employment status, there was a significant mean difference score (p≤0.001). The post hoc results showed that the employed have a statistically significant higher mean of QoL than those not employed and retired (M=12.04, SD=2.00; M=10.32, SD=1.4; M=10.48, SD=1.4; respectively). On the other hand, there was no statistical mean difference between not employed and retired (M=10.32, M=10.48, p=0.876), respectively).

In the same context, one-way ANOVA showed a statistically significant mean difference in QoL between three mean age groups (p≤0.001). The post hoc test (Schefee) showed that the participants’ age category of ≤50 years had a statistically significant higher mean of QoL than the age category of ≥61 years (M=11.44, M=10.08, p≤0.001, respectively) and age category of 51-60 years had higher mean than age category of ≥61 (M=10.84, M=10.08, p≤0.001, respectively).

## Discussion

At present, Jordan has a high incidence of Pulmonary and Cardiovascular diseases. Unfortunately, the reality of the health care systems in this country is that they provide suboptimal services that cannot provide much-needed care and attention for these patients.

The results of this study demonstrated that perceived QoL among Jordanian patients is low and reflects a poor perception of QoL. Similar results were reported internationally and triggered interventions to improve patients’QoL. For example, studies in South korea and Portugal have shown that the HRQoL was impaired in patients with COPD and other respiratory illnesses, and it further deteriorated with increase in disease severity. ^29^^,^^31^

The physical domain had the lowest perceived QoL. Several studies have also reported the physical environment among the domains with the lowest perceived QoL. ^11^^,^^26^^,^^32^ This domain relates mainly to the patients’ physical abilities to perform tasks, which were impaired due to shortness of breath and other symptoms of the disease. ^33^ Therefore, it would be imperative that healthcare professionals focus a good portion of their efforts on mitigating the physical effects of COPD on their patients to improve their quality of life through pre-planned and targeted interventions.

The current study also found that COPD patients are experiencing severe negative emotions in the psychological domain, such as anxiety and depression. This has led to a decreased QoL; this was consistent with the study by Lim *et al.* (2017), ^34^ who found that symptoms like anxiety and depression caused a lower level of QoL in COPD patients. A previous study in Jordan saw that the perceived QoL among COPD patients was highly related to feelings of uncertainty and anxiety. ^26^ Those feelings might have heightened during the COVID-19 pandemic as fear and anxiety from infection, and severe course of illness peaked. ^35^ Patients with COPD or similar complex and long-term conditions are vulnerable to mental health issues, ^35^ yet in Jordan, they do not receive any form of psychological support interventions. The health system in Jordan focuses on physical health rather than mental or psychological health. Therefore, this seems to be a huge gap that needs to be addressed quickly by the healthcare service planners.

Medical treatment enables COPD patients to function in daily life; in Jordan, especially during the pandemic, there is the issue of medical treatment availability. This further contributes to the lowered QoL perception. Similarly, Ciążyńska *et al.* (2020) study reported the unavailability of medical treatment for COPD patients and how that severely impacts their perceptions of QoL. ^12^

This study showed that personal and sexual relationships in the social domain were among the patient’s second mean score level of perceived QoL. Kurpas and colleagues (2016) reported that social relationships increase the QoL because patients do not experience loneliness and lack support. ^36^ However, participants in the study seem to be also struggling with assuming a regular social interaction and their personal and sexual relationships. COPD seems to have also affected this area of their life, resulting in lower perceptions of QoL. During the recent COVID19 pandemic, social interaction was limited, thus adding more challenges to COPD patients. Evidence indicated that the situation with COPD patients was worsening as many people refrained from visiting these patients to prevent COVID transmission; People were allowed to contact one another during Covid-19 by phone calls or internet. However, physical interaction was not allowed. ^37^ In addition, poor sexual relations (as part of the social domain) lead to a decrease in the QoL for these patients due to some of the symptoms of the disease, which, in turn, may reduce the quality of their sexual relationships and thus their QoL. This finding was consistent with a review study conducted by Merghati-Khoei and her colleagues. ^38^

When comparing the subgroups of the study, for example, those treated in private vs. governmental vs. educational hospitals, and those in different age groups, it was found that the QoL was different. Patients treated in private hospitals, who were non-smokers, unmarried, and employed have better QoL perceptions. These are all indicators of the main factors that may affect the perception of QoL for these patients in Jordan, probably internationally as well. ^5^^,^^11^^,^^14^^,^^15^^,^^18^^,^^33^^,^^39^ Therefore, these constitute reasonable goals for the healthcare service providers and planners to target to improve the QoL perceptions among patients with COPD.

## Conclusions

This study assessed the current state of QoL of Jordanian patients with COPD and identified the factors that affect it. The results indicated that the perceived QoL of COPD patients in Jordan is low and requires immediate interventions. The goal of the interventions should be to improve the healthcare service provided for these patients and thus their perceived QoL. The areas that may be targeted to achieve this goal include:

1. To give equal importance to the provision of a psychosocial and mental health support service to the patients.
2. Upgrade the services provided for these long-term healthcare users, as good quality service (i.e., in private hospitals) is associated with a better perception of QOL.
3. Initiate with patients smoking cessation interventions and follow its implementation strictly; this will significantly improve the patient’s QOL.
4. Coordinate with other governmental bodies to ensure these patients’ equal and appropriate employment opportunities. This will improve their QOL without exhausting them and putting a burden on their physical health.
5. Finally, provide extra support for married patients as it seems that despite the social benefits of marriage, it is also associated with additional responsibilities that may burden COPD patients and decrease their QOL.

## Limitations and generalizability

This study collected data through a valid and reliable questionnaire and from an adequate sample size. While the data collected is useful, it may lack depth and details. A qualitative approach may have yielded more useful and in-depth data.

The study is well positioned to be generalizable to the Jordanian population. While it may not be generalizable beyond that, neighboring countries with the same economical, sociocultural and health system contexts may learn from the results of this study.

## Data availability statement

### Underlying data

Harvard Dataverse: “COPD Patients’ Quality of Life and its Related Factors: A cross-sectional study of the Jordanian population”, https://doi.org/10.7910/DVN/UED6YA. ^28^

The data set contains the underlying data:

‐ English_Australian_WHOQOL-BREF.pdf: Study questionnaire
‐ COPD QOL.tab: This file contains the socio-demographic and QoL variables.

Data are available under the terms of the Creative Commons Zero “No rights reserved” data waiver ( CC0 1.0 Public domain dedication).

## Authors contributions

Enas Assaf & Angham Badarneh: Conceptualization, Data Curation, Formal Analysis, Investigation, Methodology, Original Draft Preparation; Ahmad Saifan & Nabeel Al-Yateem: Supervision, Writing – Final Draft Preparation, Writing – Review & Editing.
