# Supplementary material for: Chronic obstructive pulmonary disease patients' quality of life and its related factors: A cross-sectional study of the Jordanian population
Source: F1000Res. 2022 May 26;11:581. [Version 1] doi: 10.12688/f1000research.121783.1 (PMC9237555; doi:10.12688/f1000research.121783.1)
Supplement: Peer review history

# Peer review: Reviewer response for version 1

**DOI:** 10.5256/f1000research.133682.r139162

**Published:** 2022-06-27

**Authors:** Nermine M. Elcokany

**Affiliations:** 1 Department of Nursing, College of Applied Medical Sciences, King Faisal University, Al-Ahsa, Saudi Arabia

Overall, the information presented represents valuable information regarding chronic obstructive pulmonary disease which is a major public health problem internationally and even in Jordan as air pollution is really affecting COPD patients' quality of life. 

 The overall study is interesting, well-written, and structured.

 The authors have collected a detailed complete database on the topic mentioning the significance of the study as well as the global studies conducted on the quality of life of COPD patients with little research on the global level which really reflected the significance of the research problem.

- Introduction is well presented.
- The authors also wrote a well-implemented methodology although the sampling method is not clearly mentioned so better to clarify it.
- The results section reflects the aim of the study and is well organized. However, regression analysis will be better to find factors that affect QoL of COPD patients.
- The discussion is organized, well written, and has an excellent flow of ideas including recent references. 

I recommend approving this manuscript and further study is needed to explore other factors that affect the quality of life of COPD patients.

Is the work clearly and accurately presented and does it cite the current literature?

Yes

If applicable, is the statistical analysis and its interpretation appropriate?

Yes

Are all the source data underlying the results available to ensure full reproducibility?

Yes

Is the study design appropriate and is the work technically sound?

Yes

Are the conclusions drawn adequately supported by the results?

Yes

Are sufficient details of methods and analysis provided to allow replication by others?

Yes

Reviewer Expertise:

Critical Care & Emergency Nursing

I confirm that I have read this submission and believe that I have an appropriate level of expertise to confirm that it is of an acceptable scientific standard.

# Peer review: Reviewer response for version 1

**DOI:** 10.5256/f1000research.133682.r139164

**Published:** 2022-06-14

**Authors:** Ibrahim Bashayreh

**Affiliations:** 1 Fatima College, Abu Dhabi, United Arab Emirates

This study is a quantitative cross-sectional study aimed to assess COPD patients’ quality of life within the Jordanian context and identify the related factors.

 I am pretty familiar with the Jordanian context. Therefore, I agree that this topic is relevant given the high incidence of this disease in the country and the situation of the current services offered to the sufferers. The authors were also able to present these facts and establish the importance of the study in the introduction. In general terms, I found the article well written, organized, flows logically and smoothly, and is not difficult to read and understand.

 The methods used in the study are also appropriate and can achieve the aim of the study. However, the study could have been improved by adopting a mixed-methods approach, employing the different methods to understand the different dimensions of the experiences of the COPD patients. The data identified in this study is meaningful and valuable but may be limited and superficial. Therefore, I advise the authors to conduct a follow-up study further to reveal the remaining dimensions of the patients’ experiences and generate more robust findings that support the needed change in the service.

Is the work clearly and accurately presented and does it cite the current literature?

Yes

If applicable, is the statistical analysis and its interpretation appropriate?

Yes

Are all the source data underlying the results available to ensure full reproducibility?

Yes

Is the study design appropriate and is the work technically sound?

Yes

Are the conclusions drawn adequately supported by the results?

Yes

Are sufficient details of methods and analysis provided to allow replication by others?

Yes

Reviewer Expertise:

Nursing, Medical and Surgical Nursing, Critical care Nursing, Nursing education.

I confirm that I have read this submission and believe that I have an appropriate level of expertise to confirm that it is of an acceptable scientific standard.

# Peer review: Reviewer response for version 1

**DOI:** 10.5256/f1000research.133682.r139163

**Published:** 2022-06-10

**Authors:** Khaldoun Hamdan

**Affiliations:** 1 Department of Acute and chronic care nursing, Faculty of Nursing, Al-Ahliyya Amman University, Amman, Jordan

**Methodology:**

1. The sampling technique used was not explicitly identified.
2. Please identify the response rate.
3. The reliability of the tool was not mentioned in the method part. Please provide supporting references.

**Results:**

- In the paragraph related to “QoL among COPD patients” it was stated: The results in Table 2 showed that the Mean for the total score of QoL was 10.68 out of a maximum possible score of 20 (SD=1.6). In the method section, you mentioned that the questionnaire consists of 26 items rated from 1 to 5, it was unclear how the maximum possible score is 20, do you mean the maximum possible TOTAL score for the item?  And if as stated for the mean how do you calculate the maximum score for the mean score? Please clarify.

**Discussion:**

1. In the first paragraph it was stated “Unfortunately, the reality of the health care systems in this country is that they provide suboptimal services that cannot provide much-needed care and attention for these patients.” It needs supporting references otherwise it will be an opinion.
2. In the second paragraph: “The results of this study demonstrated that perceived QoL among Jordanian patients is low and reflects a poor perception of QoL” please add “COPD” before patients to be more specific.
3. Please clarify how private hospitals enhance the quality of life for COPD patients, to be more understandable for the international audience.
4. Limitation: if you used a convenience sampling it should be mentioned in the limitation section

Is the work clearly and accurately presented and does it cite the current literature?

Yes

If applicable, is the statistical analysis and its interpretation appropriate?

Yes

Are all the source data underlying the results available to ensure full reproducibility?

Yes

Is the study design appropriate and is the work technically sound?

Yes

Are the conclusions drawn adequately supported by the results?

Yes

Are sufficient details of methods and analysis provided to allow replication by others?

Partly

Reviewer Expertise:

Critical care nursing, adult care

I confirm that I have read this submission and believe that I have an appropriate level of expertise to confirm that it is of an acceptable scientific standard, however I have significant reservations, as outlined above.
